# *Pseudomonas* Quinolone Signal-Induced Outer Membrane Vesicles Enhance Biofilm Dispersion in Pseudomonas aeruginosa

**DOI:** 10.1128/mSphere.01109-20

**Published:** 2020-11-25

**Authors:** Adam C. Cooke, Catalina Florez, Elise B. Dunshee, Avery D. Lieber, Michelle L. Terry, Caitlin J. Light, Jeffrey W. Schertzer

**Affiliations:** a Department of Biological Sciences, Binghamton University, Binghamton, New York, USA; b Binghamton Biofilm Research Center, Binghamton University, Binghamton, New York, USA; c First-year Research Immersion Program, Binghamton University, Binghamton, New York, USA; d Summer Research Immersion Program, Binghamton University, Binghamton, New York, USA; University of Iowa

**Keywords:** PQS, *Pseudomonas aeruginosa*, biofilms, dispersion, outer membrane vesicles, quorum sensing, secretion systems

## Abstract

Bacterial biofilms are major contributors to chronic infections in humans. Because they are recalcitrant to conventional therapy, they present a particularly difficult treatment challenge. Identifying factors involved in biofilm development can help uncover novel targets and guide the development of antibiofilm strategies. Pseudomonas aeruginosa causes surgical site, burn wound, and hospital-acquired infections and is also associated with aggressive biofilm formation in the lungs of cystic fibrosis patients. A potent but poorly understood contributor to P. aeruginosa virulence is the ability to produce outer membrane vesicles (OMVs). OMV trafficking has been associated with cell-cell communication, virulence factor delivery, and transfer of antibiotic resistance genes. Because OMVs have almost exclusively been studied using planktonic cultures, little is known about their biogenesis and function in biofilms. Several groups have shown that *Pseudomonas* quinolone signal (PQS) induces OMV formation in P. aeruginosa. Our group described a biophysical mechanism for this and recently showed it is operative in biofilms. Here, we demonstrate that PQS-induced OMV production is highly dynamic during biofilm development. Interestingly, PQS and OMV synthesis are significantly elevated during dispersion compared to attachment and maturation stages. PQS biosynthetic and receptor mutant biofilms were significantly impaired in their ability to disperse, but this phenotype was rescued by genetic complementation or exogenous addition of PQS. Finally, we show that purified OMVs can actively degrade extracellular protein, lipid, and DNA. We therefore propose that enhanced production of PQS-induced OMVs during biofilm dispersion facilitates cell escape by coordinating the controlled degradation of biofilm matrix components.

**IMPORTANCE** Treatments that manipulate biofilm dispersion hold the potential to convert chronic drug-tolerant biofilm infections from protected sessile communities into released populations that are orders-of-magnitude more susceptible to antimicrobial treatment. However, dispersed cells often exhibit increased acute virulence and dissemination phenotypes. A thorough understanding of the dispersion process is therefore critical before this promising strategy can be effectively employed. *Pseudomonas* quinolone signal (PQS) has been implicated in early biofilm development, but we hypothesized that its function as an outer membrane vesicle (OMV) inducer may contribute at multiple stages. Here, we demonstrate that PQS and OMVs are differentially produced during Pseudomonas aeruginosa biofilm development and provide evidence that effective biofilm dispersion is dependent on the production of PQS-induced OMVs, which likely act as delivery vehicles for matrix-degrading enzymes. These findings lay the groundwork for understanding OMV contributions to biofilm development and suggest a model to explain the controlled matrix degradation that accompanies biofilm dispersion in many species.

## Abstract (precis)

Treatments that manipulate biofilm dispersion hold the potential to convert chronic drug-tolerant biofilm infections from protected sessile communities into released populations that are orders-of-magnitude more susceptible to antimicrobial treatment. However, dispersed cells often exhibit increased acute virulence and dissemination phenotypes.

## INTRODUCTION

It has long been appreciated that biofilms contribute to a majority of bacterial infections (1–4). Biofilm cells differ from planktonic cells in phenotype (5), gene expression (6), and protein production (7–10). These differences provide biofilm cells enhanced tolerance to antibiotics and host defenses (11–14). Pseudomonas aeruginosa is a clinically relevant and highly studied model organism for biofilm development. Surface-attached P. aeruginosa biofilms develop in a stepwise fashion, in which bacteria first reversibly and then irreversibly attach to a surface (7). The maturation phase is marked by the emergence of three-dimensional microcolonies during maturation I and the formation of mushroom-like clusters during maturation II (7). In response to external or endogenous cues, the final phase is initiated when bacterial cells erupt from the biofilm and disperse (7). During dispersion, motile bacteria degrade the extracellular polymeric matrix that encases them, colonize new surfaces, and recommence the biofilm life cycle (7, 15). Identification of the factors that regulate biofilm development is essential for the creation of novel therapeutics against these recalcitrant bacterial communities.

Quorum signaling is known to regulate P. aeruginosa biofilm formation (7, 16). Specifically, the Las system controls the progression from reversible to irreversible attachment (16), and the Rhl system controls the transition from irreversible attachment to maturation I (7). The *Pseudomonas* quinolone signal (PQS) has also been proposed to regulate biofilm development (17, 18). Production of PQS is initiated by the Las system through direct activation of the genes encoding the PQS regulator PqsR (18, 19) and the biosynthetic FAD-dependent monooxygenase PqsH (20, 21). PQS controls the production of many virulence factors (17), including elastase, pyocyanin (22), and iron chelators (23–25). It has been reported that PQS biosynthetic mutants are deficient in the formation of mushroom-shaped microcolonies, which are characteristic of mature biofilms (26, 27). Several hypotheses aim to connect the contributions of PQS in biofilm development to its functionality as a cell-to-cell communication signal. Rampioni and coworkers (28) suggested that PQS controls biofilm development via PqsE-dependent signaling, activating the Rhl system and its downstream effectors. It has also been shown that extracellular DNA (eDNA) contributes to biofilm maturation and that PQS-induced prophage activation results in DNA release into the biofilm (26). The buildup of 2-*n*-heptyl-4-hydroxyquinoline *N*-oxide (HQNO), which is controlled by PQS signaling, likewise results in autolysis, eDNA release, and increased biofilm biomass (29). We were interested in exploring whether other well-documented functions of PQS may also play a role during the various stages of biofilm development.

In addition to its role as a signaling molecule, PQS is also known to modulate production of outer membrane vesicles (OMVs) (30–34). OMVs are spherical structures derived from the outer membranes of Gram-negative bacteria that range from 50 to 300 nm in diameter (35–38). These nanostructures form a dedicated transport system that helps deliver cell-to-cell communication signals (30, 39, 40), nucleic acids (41, 42), proteases (43, 44), antibiotic-degrading enzymes (45, 46), lytic enzymes (47–49), iron chelators (23–25), and antibiotic resistance genes (50). In conjunction with their function as transport machinery, OMVs have also been associated with biofilm development in Helicobacter pylori (51), Vibrio cholerae (52), and Pseudomonas putida (53). Little is known about the roles that OMVs play in P. aeruginosa biofilms. However, it has been reported that OMVs are commonly found within biofilms produced by this organism (44, 54) and that their production is controlled by PQS (55).

PQS induces OMV production through a biophysical mechanism that is driven by favorable interactions with lipopolysaccharide (LPS) in the outer leaflet of the outer membrane (OM) (32, 56). These interactions promote asymmetric expansion of the outer membrane, which induces membrane curvature and ultimately leads to the production of OMVs (33). The importance of PQS in OMV production is evident from many experiments involving deletions in early biosynthetic genes (e.g., *pqsA*, coding for the anthranilate coenzyme ligase responsible for the first step in alkyl-quinolone biosynthesis [57–59]), late biosynthetic genes (e.g., *pqsH*, coding for the flavin-dependent monooxygenase responsible for the final step in PQS biosynthesis [20, 21, 60, 61]), and the PQS receptor (*pqsR*) (19, 62). Deletion of any of these genes results in drastic reductions or outright abrogation of OMV biogenesis in planktonic cultures. Our recent work demonstrated that loss of PQS production also compromised OMV production in P. aeruginosa biofilms (55). Importantly, use of these well-characterized mutants (in addition to others such as *pqsE* mutants) can help detangle the biophysical roles of PQS from its role as a signaling molecule as well as clarify contributions directly related to PQS from those of other related alkyl-quinolones.

While several studies have implicated PQS in the development of P. aeruginosa biofilms, it is not known if PQS is involved at all stages of biofilm formation. Additionally, it remains unclear if PQS affects biofilm development due to its role in quinolone signaling, virulence factor production, OMV biogenesis, or any combination of these. Here, we report that PQS and OMVs are maximally produced during biofilm dispersion. We further demonstrate that PQS biosynthetic and receptor mutants are deficient in dispersion compared to the wild type. The identified dispersion deficiency was rescued in a PQS receptor mutant through addition of exogenous PQS, supporting the notion that a signaling-independent function of PQS (i.e., OMV induction) is a major contributing factor to P. aeruginosa biofilm dispersion. We also demonstrate that purified OMVs possess protease, lipase, and nuclease activities. These results indicate that OMVs may contribute to biofilm dispersion by trafficking enzymes capable of breaking down major matrix components. Through this work, we propose a novel role of outer membrane vesicles: the enhancement of biofilm dispersion.

## RESULTS

### PQS production is elevated during dispersion.

**FIG 1:**
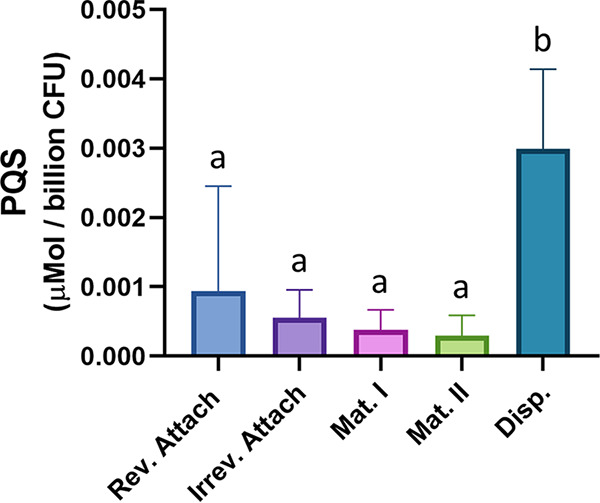
PQS production is elevated during dispersion. PQS was extracted from biofilm tube reactors grown to each of the five stages of development. Measured PQS production was normalized to micromoles per billion CFU. Error bars represent the standard deviations calculated from at least three biological replicates. Statistical significance was assessed by one-way ANOVA followed by Tukey’s *post hoc* test. Lowercase letters above the bars represent significance. Differences between bars that do not share a letter are statistically significant (*P* < 0.05).

Although OMVs are ubiquitous in P. aeruginosa biofilms (44, 55), their roles and importance in the development of a biofilm remain to be elucidated. PQS is known to promote OMV biogenesis through a biophysical mechanism (30–33), and its synthesis and export are strong indicators of OMV production potential in P. aeruginosa (34). The production of PQS is tightly regulated by quorum signaling systems (17, 21, 62, 63) and environmental conditions, such as oxygen availability (61). Due to the heterogeneous nature of biofilm development (64, 65), we hypothesized that PQS-induced OMV production would vary during biofilm progression as nutrient and substrate availability change. Using a continuous flow model, we set out to quantify total PQS production during each stage of biofilm development. Growth stages were determined via microscopic imaging of flow cells using parameters determined by Sauer and coworkers (7). In our system, reversible attachment, irreversible attachment, maturation I, and maturation II were established to occur at 8 h, 24 h, day 3, and day 5, respectively. Dispersion was induced on day 5 through exogenous addition of the native dispersion cue *cis*-2-decenoic acid (*cis*-DA). Although a P. aeruginosa biofilm will naturally produce *cis*-DA and disperse (66), we administered this molecule exogenously in order to synchronize the dispersion event (66, 67). With this study, we found that the highest level of PQS per cell was produced during dispersion (Fig. 1). Concentrations of PQS were normalized to total CFU, and a significant increase in PQS was observed during dispersion compared to that during all other biofilm stages.

### OMV production varies during biofilm development.

**FIG 2:**
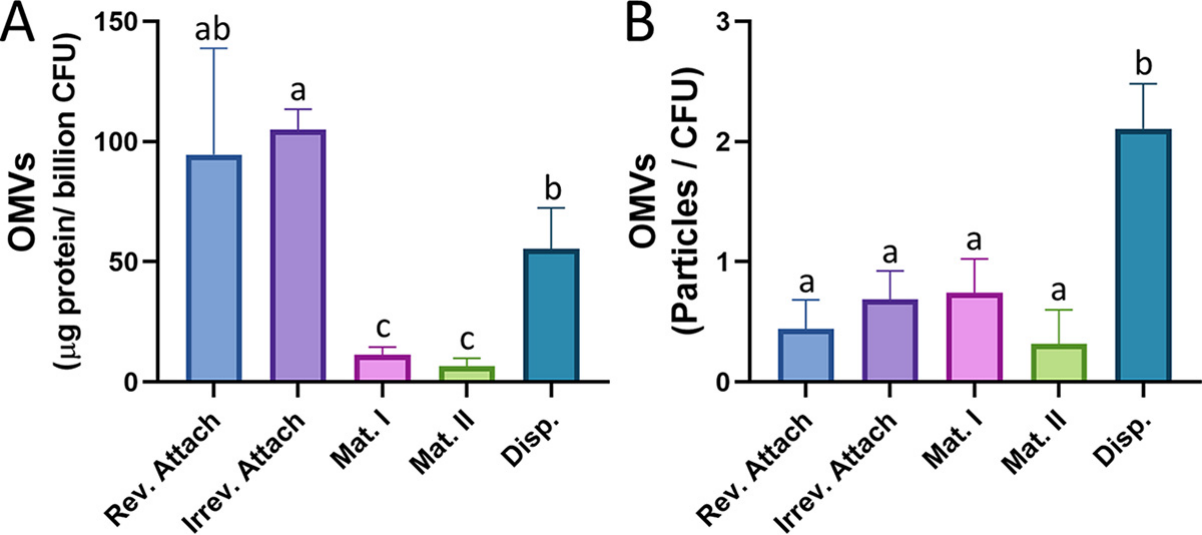
OMV production varies across biofilm developmental stages. OMVs were harvested from each stage of biofilm development and quantified using two different methods. (A) Purified OMVs were quantified by the modified Lowry assay and normalized to micrograms protein per billion CFU. (B) Purified OMVs were also quantified using nanoparticle tracking and normalized to CFU. Error bars represent the standard deviations calculated from at least three biological replicates. Statistical significance was assessed by one-way ANOVA followed by Tukey’s *post hoc* test. Lowercase letters above the bars represent significance. Differences between bars that do not share a letter are statistically significant (*P* < 0.05).

Following quantification of PQS, OMVs were isolated from the five different biofilm stages and quantified using two independent techniques: OMV protein quantification and nanoparticle tracking analysis (NTA). Modified Lowry assays showed that the highest protein levels were detected in OMV preparations harvested during reversible attachment, irreversible attachment, and dispersion (Fig. 2A). Protein concentrations in OMV pellets were normalized per billion CFU. Quantification via nanoparticle tracking analysis (which counts OMV particles directly) demonstrated that OMV production per cell remained low until the dispersion stage.

Both quantification techniques showed significantly larger numbers of OMVs present during the dispersion stage than during the maturation stages. The high level of OMV production during dispersion paralleled enhanced PQS synthesis during this stage. Interestingly, an increase in OMV production during attachment was observed via protein quantification but not via NTA.

### PQS mutants are not deficient in reversible or irreversible attachment.

**FIG 3:**
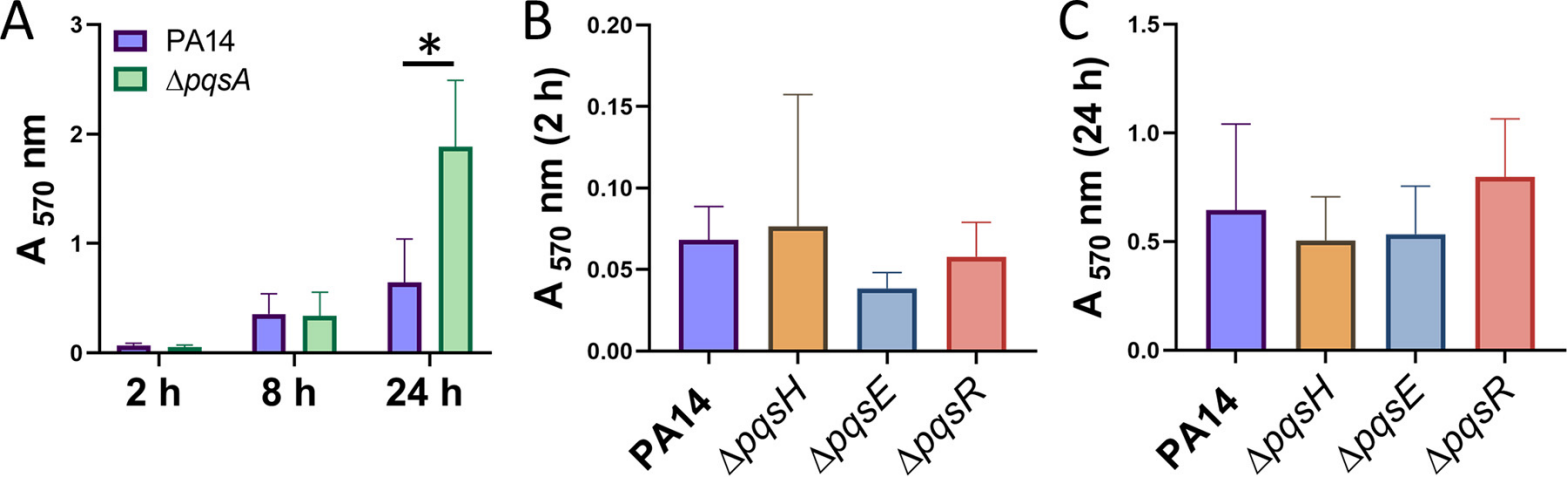
PQS mutants are not deficient in reversible or irreversible attachment. Cultures were grown in 96-well plates, planktonic cells were removed, and attached biomass was quantified using crystal violet staining. (A) PA14 and Δ*pqsA* strains were grown for 2, 8, and 24 h. (B and C) PA14, Δ*pqsH*, Δ*pqsE*, and Δ*pqsR* strains were grown for 2 h (B) and 24 h (C). Error bars represent the standard deviations calculated from a minimum of three biological replicates. Statistical significance was determined using Student's two-tailed *t* tests for panel A and one-way ANOVA for panels B and C. *, *P* < 0.05.

To determine if PQS and/or PQS-controlled phenotypes are involved in the initial stages of P. aeruginosa biofilm development, we assessed reversible and irreversible attachment abilities of wild type PA14, Δ*pqsA*, Δ*pqsH*, Δ*pqsE*, and Δ*pqsR* strains. Crystal violet attachment assays (see Materials and Methods) were performed at 2 h, 8 h, and 24 h; the former two time points were representative of reversible attachment and the latter was representative of irreversible attachment (7). We found that the Δ*pqsA* mutant was not deficient in attachment after 2 or 8 h (Fig. 3A), suggesting that quinolones are not involved in reversible attachment. Interestingly, we found that the Δ*pqsA* mutant displayed increased attachment after 24 h (Fig. 3A). These results indicate that in the wild-type strain, synthesis of at least one quinolone molecule results in reduced irreversible attachment. Next, we wanted to determine if the observed phenotypes were specifically due to the lack of PQS and PQS-mediated functions. In addition to that of the Δ*pqsA* mutant, which is blocked in production of all alkyl-quinolones, we also quantified attachment of the Δ*pqsH* mutant, which is deficient in synthesis of PQS only (20, 61). We observed no difference in attachment after 2 h or 24 h in this mutant (Fig. 3B and C). Next, we assessed attachment ability of Δ*pqsE* and Δ*pqsR* mutants, which are unable to induce Rhl-dependent virulence factors (68, 69) and respond to PQS (19), respectively. Reversible (Fig. 3B) and irreversible (Fig. 3C) attachment were unaffected in both mutants. These results indicate that PQS and PQS-mediated phenotypes do not contribute to the attachment of P. aeruginosa to an abiotic surface.

### Δ*pqsA* mutant displays diminished biofilm dispersion.

**FIG 4:**
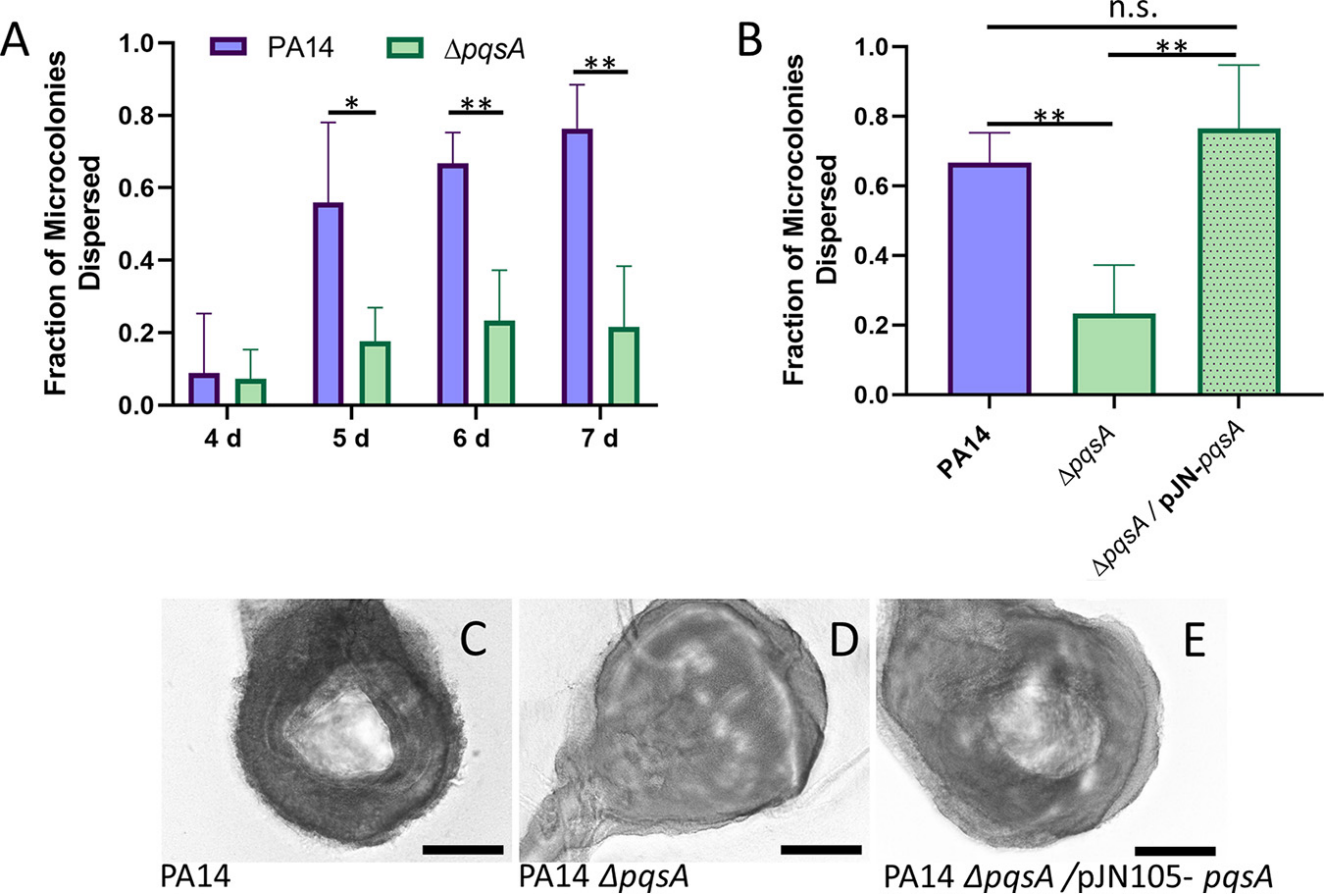
P. aeruginosa dispersion is dependent on quinolone biosynthesis. Biofilms were grown in semibatch cultures in 24-well plates, and the fraction of microcolonies that had dispersed was determined. (A) PA14 wild type and *pqsA* mutant biofilms were assessed for dispersion after 4, 5, 6, and 7 days of growth. (B) Dispersion of the *pqsA* mutant overexpressing the *pqsA* gene was assessed after 6 days of growth and compared to that of the wild type and *pqsA* mutant. Representative images show microcolonies in PA14 wild-type (C), PA14 Δ*pqsA* (D), and PA14 Δ*pqsA*/pJN105-*pqsA* (E) biofilms after 6 days of growth. Central voids are clearly visible in panels C and E. Error bars represent the standard deviations calculated from at least three biological replicates. Scale bars, 100 μm. Statistical significance was determined using Student’s two-tailed *t* test for panel A and one-way ANOVA followed by Tukey’s *post hoc* test for panel B. n.s., *P* > 0.5; *, *P* < 0.05; **, *P* < 0.01.

Our initial analysis of PQS and OMV production during biofilm development revealed high-level synthesis of both products during the dispersion phase. To determine if PQS-mediated functions are involved in this stage of development, we quantified dispersion in semibatch biofilms grown in 24-well plates. On days 4, 5, 6, and 7 after inoculation, microcolonies were observed using light microscopy, and the fraction of microcolonies that had formed central voids, a phenotypic hallmark of the dispersion process in P. aeruginosa (7, 9, 67), was determined for PA14 wild-type biofilms and for PA14 Δ*pqsA* biofilms. On day 4, little to no dispersion occurred in either strain (Fig. 4A). On days 5, 6, and 7, however, we noted significant differences in microcolony dispersion between the wild-type and Δ*pqsA* biofilms (Fig. 4A). For subsequent analyses, biofilms were grown until day 6 and analyzed for dispersion. Expression of *pqsA* in *trans* was able to restore dispersion to wild-type levels (Fig. 4B to E).

### P. aeruginosa dispersion is dependent on PQS biosynthesis but not PqsE.

**FIG 5:**
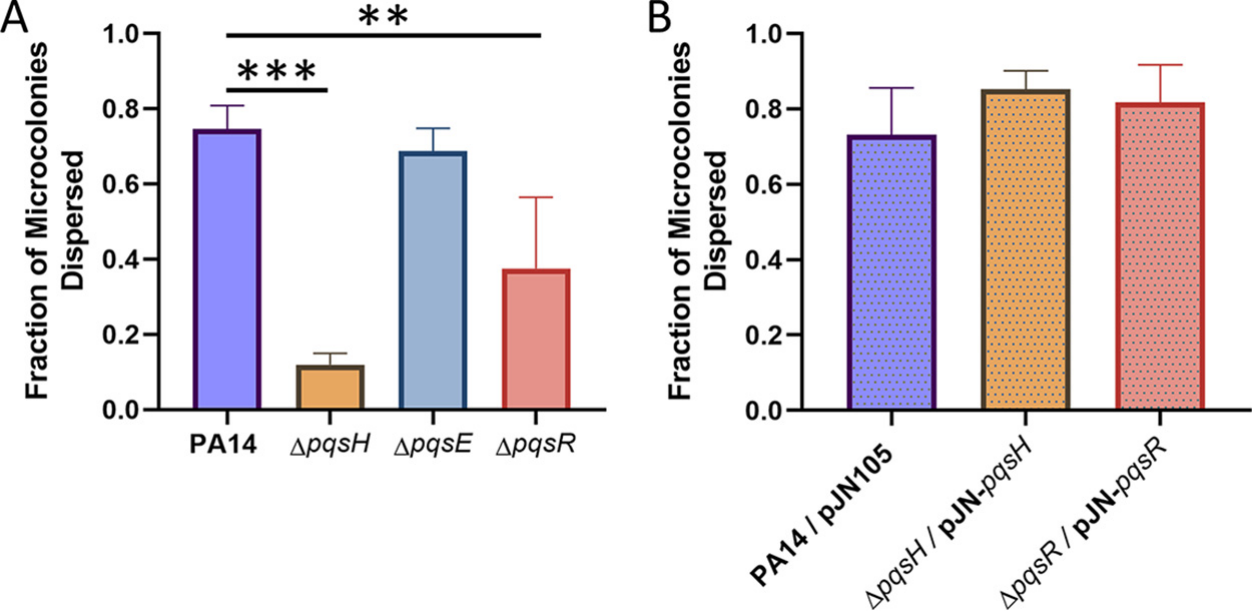
Production of PQS specifically restores native biofilm dispersion. Biofilms were grown in semibatch cultures in 24-well plates for 6 days. (A) The fraction of microcolonies dispersed was found for PA14 wild-type biofilms as well as Δ*pqsH*, Δ*pqsE*, and Δ*pqsR* biofilms. (B) Overexpression of the missing genes in the mutant backgrounds restored the dispersion that was diminished in Δ*pqsH* and Δ*pqsR* biofilms. Bars represent the standard deviations calculated from at least three biological replicates. Statistical significance was analyzed by one-way ANOVA followed by Dunnett’s *post hoc* test. **, *P* < 0.01; ***, *P* < 0.001.

The *pqsA* mutant is incapable of producing any of the secreted alkyl-quinolone compounds that depend on the *pqsABCD* operon for synthesis (e.g., 4-hydroxy-2-heptylquinoline [HHQ], PQS, HQNO, 2,4-dihydroxyquinoline [DHQ], etc.) (20, 70). For this reason, we were not able to conclude whether the inhibition of dispersion was due to a lack of PQS or a lack of one of the other quinolone molecules. To address this ambiguity, we investigated native dispersion in a *pqsH* mutant, which is blocked in production of PQS only (19, 71). Our results showed that the Δ*pqsH* mutant was deficient in dispersion compared to the wild type (Fig. 5A). The percentage of microcolonies containing voids in wild-type biofilms was 74.68% ± 6.15% compared to 11.91% ± 3.08% in the Δ*pqsH* mutant, suggesting that PQS is specifically responsible for this phenotype (Fig. 5A). However, as PQS is independently involved in both signaling (17) and OMV formation (30, 33, 34), it is unknown whether one or both of these processes are responsible for native levels of dispersion. To differentiate between these two possibilities, we investigated dispersion of a *pqsE* mutant, which produces wild-type levels of PQS (20, 21) and OMVs (data not shown) but is deficient in the production of many quorum sensing-dependent virulence factors (20). We found that the percentage of microcolonies containing voids in biofilms formed by Δ*pqsE* was 68.69% ± 6.10%, indicating that it disperses at wild-type levels (Fig. 5A). This suggests that a non-signaling-dependent function of the PQS system, such as OMV production, is likely responsible for the diminished dispersion phenotype in the Δ*pqsA* and Δ*pqsH* mutants. We also investigated dispersion in the Δ*pqsR* mutant, which displays reduced production of both PQS and OMVs (21, 30). The percentage of microcolonies containing voids in biofilms formed by the Δ*pqsR* mutant was 37.48% ± 18.97%, which is significantly lower than for the wild type (Fig. 5A). The dispersion of the Δ*pqsH* and the Δ*pqsR* mutants was restored to wild-type levels through genetic complementation (Fig. 5B). These data suggest that PQS-induced OMV production plays a significant role in P. aeruginosa biofilm dispersion.

### Exogenous PQS restores dispersion in the Δ*pqsR* mutant.

**FIG 6:**
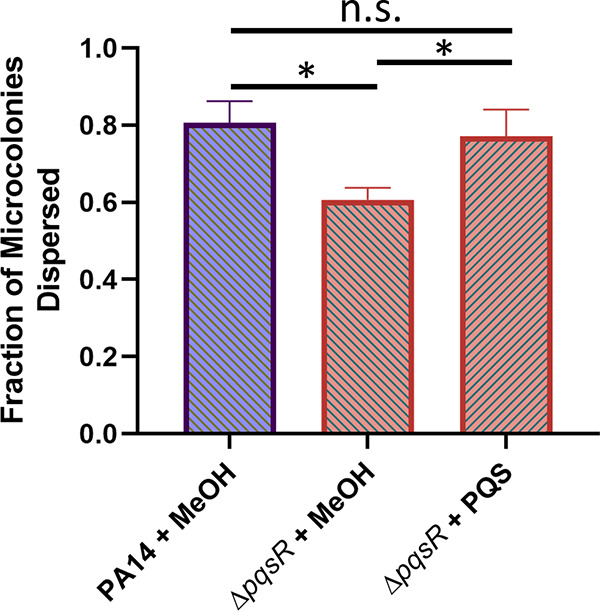
Exogenous PQS rescues Δ*pqsR* dispersion defect. PA14 wild-type and Δ*pqsR* biofilms were grown in semibatch cultures in 24-well plates for 4 days. For the following 2 days, the medium was exchanged every 12 h with fresh medium containing 40 μM PQS (+ PQS) or an equivalent amount of methanol (+ MeOH, vehicle control). Dispersion efficiency was then quantified for the strains under each condition. Error bars represent the standard deviations calculated from at least three biological replicates. Statistical significance was analyzed by ANOVA followed by Tukey’s *post hoc* test. n.s., *P* > 0.5; *, *P* < 0.05.

To confirm whether PQS modulates dispersion through an OMV-dependent mechanism, exogenous PQS was administered to a Δ*pqsR* biofilm, and dispersion efficiency was quantified. The Δ*pqsR* mutant lacks the PQS receptor and is therefore “deaf” to PQS quorum signaling (58). In contrast, PQS-induced OMV production has been shown to be driven by a biophysical mechanism that is not signaling dependent (31–33). The exogenous addition of PQS to a Δ*pqsR* biofilm restored dispersion to wild type levels (Fig. 6). These results indicate that PQS modulates dispersion using an OMV-dependent mechanism that is separate from the PQS signaling network.

### OMVs contain enzymes capable of degrading the biofilm matrix.

**FIG 7:**
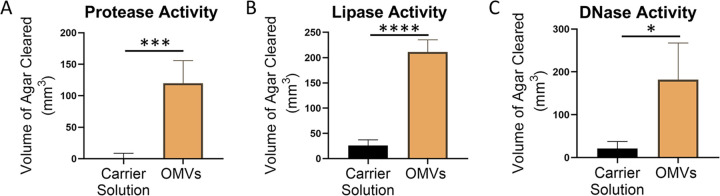
Purified OMVs display EPS-degrading activities. OMVs were harvested, washed with and resuspended in MV buffer, and added to wells punched into different types of agar. (A) Skim milk agar was used to assess protease activity. (B) Tributyrin agar was used to assess lipase activity. (C) DNase agar was used to assess DNase activity. Error bars represent the standard deviations calculated from three biological replicates. Significance was assessed using Student's two-tailed *t* tests. *, *P* < 0.05; ***, *P* < 0.001; ****, *P* < 0.0001.

Together, our results indicate that PQS-induced OMVs contribute to the dispersion of P. aeruginosa biofilms; however, the exact role the vesicles play during this developmental stage is unknown. Various studies have demonstrated that degradation of extracellular polymeric substances (EPS) of the biofilm matrix, such as polysaccharides, proteins, glycolipids, and eDNA, is a requirement for dispersion (reviewed in reference 15). Degradative enzyme activity toward these matrix components has been shown to induce dispersion in both Gram-positive and Gram-negative organisms (15, 72–78). Previous OMV proteomic analyses have identified several proteins packaged within vesicles that were predicted to have degradative activity (79, 80). Therefore, we hypothesized that OMVs may contribute to dispersion through EPS degradation. To test this hypothesis, we assessed whether purified P. aeruginosa OMVs were capable of degrading skim milk, tributyrin, and DNA to assess protease, lipase, and DNase activity, respectively. In order to acquire sufficient material for these analyses, planktonic OMVs were used. Addition of OMVs to skim milk agar resulted in the formation of a 119.8 ± 36.1-mm^3^ zone of clearing, while the addition of vehicle control (MV buffer [50 mM Tris, 5 mM NaCl, 1 mM MgSO_4_, pH 7.4] only) to skim milk agar resulted in the formation of a 0.1 ± 8.6-mm^3^ zone of clearing (Fig. 7A). This suggests that OMVs contain enzymes that have protease activity. The addition of OMVs to tributyrin agar resulted in the formation of a 211.1 ± 24.1-mm^3^ zone of clearing versus the vehicle control that produced a 25.9 ± 11.2-mm^3^ zone of clearing (Fig. 7B). This suggests that OMVs also contain enzymes that have lipase activity. Finally, the addition of OMVs and vehicle control to DNase agar resulted in the formation of 182.1 ± 85.5-mm^3^ and 21.3 ± 16.3-mm^3^ zones of clearing, respectively (Fig. 7C). This indicates that OMVs carry enzymes with DNase activity. Overall, these data support the idea that OMVs contribute to biofilm dispersion by packaging and delivering enzymes with EPS-degrading abilities.

## DISCUSSION

The present study set out to elucidate the role of PQS-induced OMV production in P. aeruginosa during biofilm development. PQS is an excellent predictor of OMV production (30, 34), and studies have consistently shown that inhibition of PQS synthesis (whether genetic or environmental) results in dramatic reduction of OMV formation (30, 55, 61). Although extracellular vesicles have been observed in the absence of PQS (54, 55), their origins and composition are uncertain, and they are frequently mixed-composition vesicles resulting from cellular disintegration. For this reason, we were surprised to measure high levels of OMVs during reversible and irreversible attachment using protein-based quantification, despite low PQS concentrations (Fig. 1 and 2). High levels of OMV production during these initial stages measured by Lowry assay were not corroborated by nanoparticle tracking analysis, suggesting that the protein detected in these OMV preparations was not representative of OMV concentration but likely the result of non-OMV-related protein components. As a result, we predicted that PQS and OMVs were not significant effectors of reversible and irreversible attachment. This notion was supported by our crystal violet attachment assays, which demonstrated that Δ*pqsA*, Δ*pqsH*, Δ*pqsR*, and Δ*pqsE* mutants had wild-type levels of reversible attachment (Fig. 3). It is notable, however, that several studies have identified an increase in biofilm formation when OMV production is stimulated (22, 51, 81, 82). Kang et al. (23) described that *pqsA*, but not *pqsH* or *pqsE*, was required for early biofilm attachment under static conditions. Others have reported that PQS and, possibly, OMVs are more important in later maturation stages (26, 27, 83). In contrast, Ionescu et al. showed in Xylella fastidiosa that OMV production inhibited bacterial attachment to plant surfaces (84). In the face of these conflicting reports, it is interesting that we found that the *pqsA* mutant had increased irreversible attachment versus that of the wild type at 24 h (Fig. 3A). During early biofilm development, attachment is required. Therefore, it might be beneficial for P. aeruginosa to reduce PQS production at this time to avoid the potential interference of PQS-induced OMVs with cell attachment. Regardless, it is evident that the role of OMVs in early-stage biofilm development remains unclear and will require further studies to elucidate.

During maturation I and II, we saw that both PQS and OMV production were relatively low (Fig. 1 and 2). Allesen-Holm et al. proposed that PQS-induced prophage-mediated cell lysis results in eDNA release and the development of a three-dimensional microcolony architecture (26). A separate study by Tettmann et al. showed that enzymatic degradation of PQS resulted in increased iron availability and enhanced biofilm formation for early and mature biofilms (85). The latter report aligns with our observations and offers an explanation as to why cells might reduce PQS production during biofilm maturation. It is important to note that although PQS production was reduced during maturation in our study, it was not eliminated. The same was true for OMV production. It is likely that baseline levels of PQS are important for PQS-mediated cell lysis and eDNA release, while reduced numbers of OMVs may carry out structural or transportation roles. At this developmental stage, elevated levels of PQS and PQS-induced OMVs could even have negative effects on biofilm development, as OMVs have been predicted to contain degradative enzymes (79, 80), which could break down major components of the EPS.

While our results suggest that PQS and OMVs may play only minor (or undetermined) roles during attachment and maturation, they highlight a major increase in production of both factors upon the initiation of biofilm dispersion (Fig. 1 and 2). This observation led us to speculate that PQS and PQS-induced OMVs are important for proper dispersion of P. aeruginosa biofilms. To test this hypothesis, we analyzed microcolony dispersion frequencies for four mutants: Δ*pqsA*, Δ*pqsH*, Δ*pqsR*, and Δ*pqsE.* Biosynthetic (*pqsA* and *pqsH*) and receptor (*pqsR*) mutants dispersed at much lower frequencies than the wild type (Fig. 4 and 5). Because the Δ*pqsA* (cannot produce any alkyl-quinolones) and Δ*pqsH* (produces all alkyl-quinolones except PQS) mutants were similarly impaired in dispersion, we can conclude with confidence that PQS, specifically, is required (i.e., not HHQ, HQNO, DHQ, or any of the other alkyl-quinolones lost in the Δ*pqsA* mutant). Rescue of the Δ*pqsR* phenotype by exogenous PQS demonstrated that the physical presence of PQS was required rather than signaling through its receptor (Fig. 6). The importance of a nonsignaling function of PQS is further supported by the fact that the *pqsE* mutant showed no deficiency in dispersion, confirming that signaling downstream of PqsR is also not involved in this phenotype (Fig. 5). Together, these results strongly suggest that PQS modulates P. aeruginosa dispersion in a signaling-independent manner (e.g., by inducing OMV production).

Degradation of the extracellular matrix that encapsulates biofilm cells is a fundamental requirement for dispersion (15), and enzymes with matrix-degrading activity have been described to induce dispersion in mature biofilms in several organisms (15, 73–78, 86). Here, we report that purified OMVs possess protease, lipase, and DNase activity (Fig. 7). These results are consistent with early studies showing some of the same activities associated with OMVs (44, 47, 48, 87). A recent study by Esoda and Kuehn found that OMVs traffic the P. aeruginosa peptidase PaAP and can deliver the peptidase to 1-h-old P. aeruginosa and K. pneumoniae biofilms grown on A549 tissue culture cells, resulting in decreased biofilm biomass (88). Others have provided evidence that proteases are required for dispersion in Staphylococcus aureus biofilms (73) and P. putida biofilms (75). In P. aeruginosa, eDNA degradation has been shown to result in biofilm disaggregation (26, 89), and recent work by Cherny and Sauer showed that eDNA degradation is required for dispersion of P. aeruginosa (74). In Propionibacterium acnes, secreted lipases have also been demonstrated to enhance the dispersion response (90). Delivery of these degradative enzymes using OMVs may increase the enzymes’ efficacy, facilitate specific targeting to sites of degradation, and reduce potential deactivation of the enzymes while in transit. Bomberger et al. demonstrated that the cystic fibrosis transmembrane conductance regulator (CFTR) inhibitory factor (Cif) produced by P. aeruginosa was orders-of-magnitude more potent when delivered within OMVs (91). We therefore propose that PQS-induced OMVs enhance biofilm dispersion by delivering and potentially enhancing the activity of enzymes required for EPS degradation.

Consistent with a role for OMVs in biofilm degradation, a number of groups have previously shown that the addition of OMVs to established biofilms can result in a reduction of biofilm biomass (88, 92). However, specific follow-up experiments (88) went on to confirm that such biomass “disruption” arose from disorganized sloughing of large chunks of biofilm and was not analogous to biofilm dispersion in terms of overall mechanism or in the phenotypic profile of the released cells. Natural biofilm OMVs do not enter from outside but rather are produced within the microcolonies. Knowing this, we reasoned that the provision of exogenous PQS to the PQS-null Δ*pqsR* mutant biofilm would best allow for OMV biogenesis to be stimulated *in situ* within the biofilm and most accurately allow for a natural dispersion response. Satisfyingly, our method was able to demonstrate chemical complementation of the dispersion defect while also recapitulating the distinctive microcolony central voids that are characteristic of natural dispersion in P. aeruginosa rather than the wholesale “disruption” and “detachment” described in previous works using exogenous addition of preformed OMVs (88, 92).

Overall, our data support the idea that PQS-induced OMVs enhance biofilm dispersion. We conclusively demonstrated that PQS is the only alkyl-quinolone produced by P. aeruginosa that affects biofilm dispersion, and by eliminating the involvement of the well-characterized PQS signaling effectors *pqsR* and *pqsE*, we uncovered that it is a signaling-independent function of PQS that is paramount (e.g., PQS-induced OMV formation). This aligns well with our analysis showing that OMVs are highly upregulated during biofilm dispersion. Finally, we demonstrated that OMVs have the capability to breakdown extracellular DNA, lipids, and proteins—all major components of the biofilm EPS matrix. Nevertheless, it is impossible to completely rule out the involvement of as-yet-unknown or uncharacterized functions of PQS in the biofilm dispersion response. Rampioni et al. (69) reported that addition of exogenous PQS to planktonic cells can alter the expression of a subset of genes independently of *pqsR*. However, the authors discuss that these effects are almost certainly indirect, and the heavy focus of altered gene expression toward iron metabolism and general metabolic pathways point to general environmental changes playing a role in that response (perhaps due to iron chelation and aggregation of exogenously added PQS). Interestingly, it has recently been reported that iron depletion may induce biofilm dispersion through an unknown mechanism that seems to involve the extracellular polysaccharide Psl (93). PQS is known to chelate iron (23–25), and we are intrigued by this possible connection because PQS-laden OMVs have been suggested to participate in iron scavenging under some conditions (94). It is important to point out, however, that if either of these hypothesized mechanisms contribute to PQS-induced biofilm dispersion alongside OMV-mediated matrix degradation, they both still point to an important role for OMVs. Since OMVs are the well-established vehicle for PQS export and delivery, neither off-receptor signaling nor iron scavenging mechanisms could likely occur without them. Thus, PQS-induced OMVs may enhance biofilm dispersion in even more ways than we have characterized here.

## MATERIALS AND METHODS

### Strains, growth conditions, and media.

All experiments were carried out using P. aeruginosa strains described in Table 1. The Δ*pqsE* and Δ*pqsR* clean-deletion mutant strains were constructed using the pEX18gm suicide vector (95), and *pqsE* and *pqsR* were overexpressed in their respective mutant backgrounds using the pJN105 vector (96). Primer sequences used for construction of the vectors can be found in Table S1 in the supplemental material. Biofilm tube reactors were inoculated as described below. Planktonic cultures were inoculated to an optical density at 600 nm (OD_600_) of 0.01 and grown at 37°C with shaking at 250 rpm. Planktonic cultures were grown in lysogeny broth (LB) or brain heart infusion (BHI) medium. Planktonic cultures of strains carrying the pJN105 vector were grown in the presence of gentamicin (50 μg/ml), while biofilm cultures of the same strains were not.

**TABLE 1:** Bacterial strains and plasmids used in this study

Strain or plasmid	Description	Source or reference
Strains		
E. coli		
DH5α	F^−^ ϕ80*lacZ*ΔM15 Δ(*lacZYA-argF*)*U169 recA1 endA1 hsdR17* (r_K_^−^, m_K_^+^) *phoA supE44* λ^−^ *thi-1 gyrA96 relA1*	101
P. aeruginosa		
PA14	Wild-type P. aeruginosa strain	102
Δ*pqsA*	*pqsA* clean deletion in PA14 background	Kind gift of Marvin Whiteley
Δ*pqsH*	*pqsH* clean deletion in PA14 background	103
Δ*pqsE*	*pqsE* clean deletion in PA14 background	This study
Δ*pqsR*	*pqsR* clean deletion in PA14 background	This study
Plasmids		
pEX18gm	Gm^r^, suicide plasmid for gene replacement in P. aeruginosa	95
pEX18gm-*pqsE*	Gm^r^, pEX18gm-derived vector for clean-deletion of *pqsE*	This study
pEX18gm-*pqsR*	Gm^r^, pEX18gm-derived vector for clean-deletion of *pqsR*	This study
pJN105	Gm^r^, *araC-pBAD* expression vector	96
pJN105-*pqsA*	Gm^r^, pJN105-derived *pqsA* overexpression vector	55
pJN105-*pqsH*	Gm^r^, pJN105-derived *pqsH* overexpression vector	This study
pJN105-*pqsR*	Gm^r^, pJN105-derived *pqsR* overexpression vector	This study
pCR 2.1	Amp^r^, Kan^r^, TA-cloning vector	Invitrogen
pRK2013	Km^r^, helper plasmid used for triparental mating	104

10.1128/mSphere.01109-20.1 TABLE S1 Primers used in this study. Underlined sequences show recognition sites for restriction endonucleases. Download Table S1, DOCX file, 0.01 MB. Copyright © 2020 Cooke et al. 2020 Cooke et al. This content is distributed under the terms of the Creative Commons Attribution 4.0 International license.

### Biofilm growth.

Biofilms were grown in both continuous and semibatch culture systems. For continuous culture, biofilms were grown in size 14 Masterflex silicone tubing (Cole Parmer) as previously described (7, 97). Cultures were inoculated under static conditions and allowed to attach for 1 h prior to initiation of flow. Biofilms were grown at 22°C in 5% LB medium under a constant flow rate of 0.18 ml/min until the desired stage of biofilm growth: 8 h for reversible attachment, 24 h for irreversible attachment, 3 days for maturation I, and 5 days for maturation II (as determined previously [7] and in this study by microscopic flow cell images). To validate developmental stages, biofilms were grown under identical conditions in BioSurface Technologies flow cells and visualized by bright-field microscopy. Biofilms were harvested from continuous culture systems using the rolling pin method (7). Mature biofilms were collected into sterile saline (1 ml/line). For stage 5, biofilm dispersion, 5% LB with or without the native dispersion induction molecule *cis*-2-decenoic acid (310 nM) was administered to 5-day-old biofilms. Biofilms were incubated with either treated or untreated medium under static flow for 1 h (66, 67). Following induction, dispersed cells in the bulk liquid were collected under native flow, leaving attached biofilm cells behind in the tubing. To quantify if a dispersion event occurred, OD_600_ measurements were taken of the collected bulk liquid from the treated sample and compared to measurements from the untreated sample.

Semibatch biofilms for dispersion analyses were cultured in 24-well plates as previously described (67) with minor modifications. Briefly, wells were inoculated with 500 μl of culture adjusted to an OD_600_ of 0.01 in 20% LB. Plates were incubated at 37°C with shaking at 250 rpm at a 30° angle for 24 h. Medium was then replaced with 250 μl of 20% LB medium and returned to the incubator under the same conditions. Medium changes were repeated every 12 h for up to 7 days. For chemical complementation experiments, strains were inoculated and grown as described above for the first 4 days. From 4 days postinoculation to 6 days postinoculation, the medium was changed with 20% LB containing 40 μM PQS or 20% LB containing an equivalent amount of the carrier solution (methanol) every 12 h.

### PQS extraction and quantification.

PQS was extracted from biofilms harvested at each stage of development. Biofilms were homogenized to reduce aggregation, and PQS was extracted using 1:1 acidified ethyl acetate as previously described (34, 55, 61, 98). The organic phase was separated and dried under nitrogen. Samples were resuspended in optima-grade methanol and spotted onto straight-phase phosphate-impregnated thin-layer chromatography (TLC) plates that had been activated at 100°C for 1 h. PQS was visualized by intrinsic fluorescence after excitation under long-wave UV light. Digital images were captured and analyzed using a Bio-Rad ChemiDoc XRS system and Image Lab densitometry software. PQS concentration values were normalized to total CFU.

### OMV isolation and quantification.

OMVs were isolated from harvested biofilms as previously described (55). Biofilms were homogenized to reduce aggregation, and preparations were centrifuged at 16,000 × *g* for 10 min at 4°C to remove cells. The supernatant was then passed through a 0.45-μm polyethersulfone filter to remove any remaining cells. OMVs were pelleted and purified from the supernatant using a Thermo Scientific S50-A rotor (50,000 rpm for 1.5 h) and resuspended in 500 μl of sterile MV buffer (50 mM Tris, 5 mM NaCl, 1 mM MgSO_4_, pH 7.4) (34, 55).

OMVs were then quantified by both modified Lowry protein assay (Thermo) (99) and nanoparticle tracking analysis (NTA) (34, 55, 100). The modified Lowry assay was performed according to the manufacturer’s instructions. For NTA, Purified vesicles were diluted to obtain 20 to 100 particles per frame and analyzed using a Malvern NanoSight NS3000 system (camera level 12 and gain of 1) and corresponding software (NTA 3.1). NTA uses laser diffraction to monitor the movement of individual OMV particles and thus reports a direct count of particle number. Total protein and OMV particle numbers were normalized to total CFU in the original sample.

### Crystal violet attachment assays.

To assess attachment, 96-well plates were inoculated with 200 μl of culture in LB at an OD_600_ of 0.01. The plates were then incubated at 37°C shaking at 250 rpm for 2, 8, or 24 h. Biomass was quantified by crystal violet (CV) staining. Supernatant was removed from wells and replaced by 200 μl deionized (DI) water. Fifty microliters of 0.1% CV in DI water was then added to each well, and plates were incubated for 15 min at 37°C with shaking at 250 rpm. Following staining, wells were washed 4 times with DI water to remove any unattached cells and unbound CV. Plates were then blotted vigorously with a paper towel and allowed to dry. Once dry, 200 μl of 95% ethanol was added to each well, and the plate was incubated for 10 min at 37°C with shaking at 250 rpm to solubilize the CV. The absorbance of each well was then read at 570 nm.

### Assessment of dispersion phenotype in 24-well microtiter plates.

Biofilms were grown as described above for up to 7 days, and native dispersion was assessed as previously described (9, 67). Briefly, biofilm microcolonies were observed by transmitted light using an Olympus BX60 microscope and a 20× UPlanF Olympus objective. Images were captured using a ProgRes CF camera (Jenoptik, Jena, Germany) and processed with ProgRes CapturePro 2.7.7 software. Dispersion efficiency was quantified by determining the percentage of microcolonies that had developed an interior void. For each biological replicate, biofilms were grown in 2 to 4 wells of a 24-well plate, and all microcolonies that had formed in these biofilms were analyzed for dispersion. The total number of microcolonies analyzed for each strain and condition are presented in Table S2.

10.1128/mSphere.01109-20.2 TABLE S2 Number of microcolonies analyzed. The total number of microcolonies analyzed for void formation. n, number of microcolonies analyzed; +PQS, strain was grown in the presence of medium containing 40 μM PQS from 4 days of biofilm growth until 6 days; +MeOH, strain was grown in the presence of the PQS carrier solution, methanol, at a concentration equivalent to the one added under the +PQS condition. Download Table S2, DOCX file, 0.01 MB. Copyright © 2020 Cooke et al. 2020 Cooke et al. This content is distributed under the terms of the Creative Commons Attribution 4.0 International license.

### Analysis of degradative enzyme presence in OMVs.

In order to acquire enough material for enzymatic analysis, OMVs were harvested from planktonic cultures as described above. OMV preparations were quantified using NTA and diluted to 2 × 10^11^ particles/ml in MV buffer. One hundred eighty microliters of OMVs was then added to wells punched in agar using a method described previously (90). Agar plates impregnated with protein, lipid, or DNA were prepared, and wells were punched within the agar using the wide end of a 1,000-μl pipette tip. Each 100-mm-diameter petri dish used contained 25 ml of an agar solution. For proteomic analysis, milk agar plates were prepared (2.5 g/liter skim milk [BD] and 15 g/liter agar [BD]). For these plates, skim milk and agar were autoclaved separately, cooled to 50°C, and then mixed together prior to pouring plates. For lipase analysis, 50% tributyrin agar was used (11.5 g/liter tributyrin HiVeg agar base [HiMedia], 5 ml/liter tributyrin [TCI], 7.5 g/liter agar [BD]). Specifically, the agar was boiled in water, tributyrin was added, and the mixture was homogenized in a blender for approximately 20 s to ensure effective dispersal of the hydrophobic tributyrin throughout the medium. Once autoclaved, this agar was stirred while cooling to approximately 60°C, and the plates were then poured. For DNase analysis, DNase plates were prepared (21 g/liter Difco DNase test agar with methyl green [BD], 7.5 g/liter agar [BD]). After addition of OMVs to the punched wells, plates were sealed with parafilm and incubated at 37°C for 24 h prior to measuring the diameter of the zone of clearing.

### Statistical analysis.

Statistical analyses were performed as described in figure legends and carried out in GraphPad Prism 8.
