# Supplementary material for: Pseudomonas Quinolone Signal-Induced Outer Membrane Vesicles Enhance Biofilm Dispersion in Pseudomonas aeruginosa
Source: mSphere. 2020 Nov 25;5(6):e01109-20. doi: 10.1128/mSphere.01109-20 (PMC7690959; doi:10.1128/mSphere.01109-20)
Supplement: TABLE S1 [file mSphere.01109-20-st001.docx]

**Table S1. Primers used in this study.** Underlined sequences show recognition sites for restriction endonucleases.

| Primer Name | Sequence (5’ to 3’) |
| --- | --- |
| pJN105-MCS-For-Seq | TAGCGGATCCTACCTGACGC |
| pJN105-MCS-Rev-Seq | CCATTCGCCATTCAGGCTG |
| pqsE-pJN105-For | GTCAGTCAGAATTCCTGAGGAGGTGAACCGGCC |
| pqsE-pJN105-Rev | GTCAGTCAGAGCTCTCAGTCCAGAGGCAGCGC |
| pqsR-pJN105-For | GTCAGTCAGAATTCCACGCGCCACCCAATAAAAGG |
| pqsR-pJN105-Rev | GTCAGTCAGAGCTCAACGCTCTACTCTGGTGCGG |
| pqsE-KO-A | GTAGTAGAGCTCCGTTTCTTAGAACCGTCCCTAGCTC |
| pqsE-KO-B | TCAGTCCAGAGGCAGCGCGACCGGGAGCCGAAAGCC |
| pqsE-KO-C | GGCTTTCGGCTCCCGGTCGCGCTGCCTCTGGACTGA |
| pqsE-KO-D | GTAGTAGGATCCCAGTTCTGCCTGCTCGGC |
| pqsR-KO-A | GTAGTAGAGCTCCGAAGACGGACTGCTGGACC |
| pqsR-KO-B | GCGCCTTCGGGCCTGACCCTTATTCCTTTTATTGGGTGGCGC |
| pqsR-KO-C | GCGCCACCCAATAAAAGGAATAAGGGTCAGGCCCGAAGGCGC |
| pqsR-KO-D | GTAGTAGGATCCAACTGCTGGTGCTGTCGC |
