# Supplementary material for: Pseudomonas Quinolone Signal-Induced Outer Membrane Vesicles Enhance Biofilm Dispersion in Pseudomonas aeruginosa
Source: mSphere. 2020 Nov 25;5(6):e01109-20. doi: 10.1128/mSphere.01109-20 (PMC7690959; doi:10.1128/mSphere.01109-20)
Supplement: TABLE S2 [file mSphere.01109-20-st002.docx]

**Table S2. Number of microcolonies analyzed.** The total number of microcolonies analyzed for void formation. n: number of microcolonies analyzed. +PQS: strain was grown in the presence of medium containing 40 μM PQS from 4 days of biofilm growth until 6 days. +MeOH: strain was grown in the presence of the PQS carrier solution, methanol, at a concentration equivalent to the one added in the +PQS condition.

| **Strain** | **Growth (days)** | **n** |
| --- | --- | --- |
| PA14 | 4 | 42 |
| PA14 | 5 | 80 |
| PA14 | 6 | 377 |
| PA14 | 7 | 199 |
| PA14 Δ*pqsA* | 4 | 108 |
| PA14 Δ*pqsA* | 5 | 172 |
| PA14 Δ*pqsA* | 6 | 176 |
| PA14 Δ*pqsA* | 7 | 199 |
| PA14 Δ*pqsA /* pJN105-*pqsA* | 6 | 144 |
| PA14 Δ*pqsH* | 6 | 190 |
| PA14 Δ*pqsE* | 6 | 222 |
| PA14 Δ*pqsR* | 6 | 424 |
| PA14 / pJN105 | 6 | 151 |
| PA14 ΔpqsH / pJN105-*pqsH* | 6 | 192 |
| PA14 Δ*pqsR* / pJN105-*pqsR* | 6 | 155 |
| PA14 + MeOH | 6 | 167 |
| PA14 *pqsR* + MeOH | 6 | 369 |
| PA14 *pqsR* + PQS | 6 | 454 |
